# Prevalence of visceral artery involvement in patients with peripheral artery disease found on run-off MRA

**DOI:** 10.1186/s12880-021-00615-2

**Published:** 2021-06-02

**Authors:** Felix Streckenbach, Felix G. Meinel, Felix Ammermann, Anke Busse, Andreas Neumann, Thomas Heller, Marc-André Weber, Ebba Beller

**Affiliations:** 1 Institute of Diagnostic and Interventional Radiology, Pediatric Radiology and Neuroradiology, University Medical Centre Rostock, Ernst-Heydemann-Str. 6, 18057 Rostock, Germany; 2 Department of General, Thoracic, Vascular and Transplantation Surgery, University Medical Centre Rostock, Rostock, Germany; 3 Center for Transdisciplinary Neurosciences Rostock, University Medical Centre Rostock, Rostock, Germany

**Keywords:** Magnetic resonance angiography, Peripheral artery disease, Atherosclerosis, Visceral artery

## Abstract

**Background:**

In patients with peripheral artery disease (PAD), run-off MR-angiography (MRA) is a commonly performed diagnostic test to obtain high-resolution images for evaluation of the arterial system from the aorta through the distal run-off vessels. The aim of this study was to investigate the prevalence of visceral artery involvement (VAI) in patients with PAD and leg symptoms examined with run-off MRA.

**Methods:**

We retrospectively analyzed 145 patients (median age 68 years, range 27–91) who underwent MRA due to known or suspected PAD at our institution between 2012 and 2018. MRA examinations were re-evaluated for visceral artery stenosis. Patient dossiers were reviewed to determine cardiovascular risk factors, kidney function and Fontaine stage of PAD.

**Results:**

Involvement of at least one visceral artery with ≥ 50% diameter stenosis was found in 72 (50%) patients. There were no differences in age, gender, MRA indication, Fontaine stage, levels of C-reactive protein (CRP), cardiovascular risk factors or vascular comorbidities between patients with and without VAI. Renal artery (RA) involvement with ≥ 50% diameter stenosis was observed in 28 (20%) of patients. Patients with involvement of the RA were more likely to suffer from hypertension (79 vs. 54%, *p* = 0.019) and reduced renal function (glomerular filtration rate 70 vs. 88 mL/min/1.73m^2^, *p* = 0.014).

**Conclusion:**

Visceral artery stenosis can be seen in half of patients with known or suspected PAD and leg symptoms on run-off MRA. Investigating for RA stenosis in patients with PAD and hypertension and/or impaired renal function may have high diagnostic yield.

**Supplementary Information:**

The online version contains supplementary material available at 10.1186/s12880-021-00615-2.

## Background

Peripheral arterial disease (PAD) is a common condition and has increased in prevalence by 23.5% between 2000 and 2010. Because PAD is largely a disease of the elderly, this trend is likely to continue as life expectancy increases and exposure to atherogenic risk factors persist [1, 2]. Since atherosclerosis is a systemic disease, the majority of patients with PAD have concomitant atherosclerosis of different arterial beds [3]. Correspondingly, several studies have shown a strong correlation between PAD and coronary artery disease [4–6] or stroke [7, 8] and a high prevalence of carotid [9, 10] and renal artery stenosis in patients with PAD [11–13].

In patients with PAD, run-off MRA is a commonly performed diagnostic test to obtain high-resolution images for evaluation of the arterial system from the aorta through the distal run-off vessels [14, 15]. Besides the peripheral arterial vasculature and aortic bifurcation, MRA also potentially visualizes visceral arteries including celiac trunk, superior and inferior mesenterial artery (SMA and IMA) and renal and accessory renal arteries. MRA scoring systems for patients with PAD include the run-off resistance score, which is determined by multiplying individual vessel factors of the common iliac, internal iliac, external iliac, deep femoral, superficial femoral, anteriortibial, posteriortibial, peroneal and pedal arch, with the degree of occlusion, adding 1 to each segment for intrinsic resistance of a healthy segment [16] and MRA index, which is calculated by dividing the arterial tree of the leg into 16 segments from the distal aorta to the run-off vessels and using a five point ordinal scale to grade each segment [17]. Both MRA scoring systems do not take into account involvement of the visceral arteries. However, VAI is associated with an increased mortality risk [12, 18] and can result in a vicious circle of arterial hypertension and consequently more vascular damage [19].

Thus, the aim of this study was to evaluate the prevalence of visceral artery involvement in patients with known or suspected PAD and leg symptoms on MRA of the run-off vasculature.

## Methods

### Study design and ethical approval

This study was designed as a retrospective, single-center cohort study. We included adult patients with known or suspected PAD who underwent a contrast-enhanced run-off MRA at our institution between January 2012 and April 2018 for leg symptoms. We only included examinations, in which the scan range allowed for the evaluation of presence or absence and degree of stenosis of at least both renal arteries and the IMA. We excluded patients with other indications for MRA, incomplete examinations, insufficient image quality due to artifacts, examinations with a modified protocol, missing images or clinical information, and repeat investigations of identical patients. For the sub-analysis of renal artery involvement, we also excluded four patients who were status post nephrectomy.

The study protocol was approved by the responsible institutional review board (blinded) with waiver of informed consent. The study was conducted in compliance with the Declaration of Helsinki in its current form.

### Patient selection

Patients were identified by retrospective search of our radiology information system (Centricity 5.0, GE Healthcare, Barrington, Illinois). All consecutive patients meeting all inclusion criteria and none of the exclusion criteria were included in the analysis. Review of electronic patient charts was performed to determine cardiovascular risk factors, vascular comorbidities and Fontaine stage of PAD at the time of the MRA examination. The Fontaine classification is solely based on clinical symptoms and ranges from stage I to IV (I: asymptomatic, II: mild claudication pain, IIa: claudication at a distance > 200 m, IIb: < 200 m, III: rest pain, IV: necrosis and/or gangrene) [20]. CRP (local reference value < 5 mg/L), serum creatinine and estimated glomerular filtration rate (eGFR), using the Cockroft und Gault formula, were also obtained by reviewing electronic patient charts.

### MRA technique

All MRA examinations were performed with a 3 T unit (Magnetom Verio, Siemens Healthineers, Erlangen, Germany). The localizer consisted of three imaging stacks in transverse, sagittal and coronal orientation at four levels including abdomen, pelvis, upper legs/ knee and lower legs. A pre- and post-contrast 3D T1-weighted MRA sequence was acquired in coronal plane, also at four levels, using following parameters: TR 2.85–3.58 ms and TE 1.03–1.25 ms and with 0.2 mmol / kg body weight Gadobutrol (Gadovist®, Bayer Vital, Leverkusen, Germany). A time-resolved angiography with interleaved stochastic trajectories (TWIST) of the lower legs was performed between the pre- and postcontrast 3D T1-weighted MRA with the following parameters: TR 3.16 ms and TE 1.17 ms and a fixed dose of 4 ml Gadobutrol. The flow rate of 2 ml/s Gadobutrol was applied for all MRA examinations. The entire MRA datasets were archived in our PACS software (IMPAX 6.5.3, Agfa HealthCare, Bonn, Germany).

### Image analysis for the grading of visceral artery stenosis

MRA datasets of all 145 patients were re-evaluated by two experienced readers in consensus who had not been involved in the patient selection process (one radiology fellow, one board-certified radiologist with sub-specialization in cardiovascular imaging, initials blinded). Thin-section images of non-subtracted post-contrast MRA images of the abdomen were viewed in 3D multiplanar reformats using a 3D module within our PACS. In addition, subtracted MRA images of the abdomen were also viewed for the assessment of the visceral arteries.

Diameters of celiac trunk, SMA, left and right renal artery and accessory renal arteries were visually estimated and categorized on a grade 0–5 scale:

- Grade 0: no stenosis
- Grade 1: stenosis < 30%
- Grade 2: stenosis 30–49%
- Grade 3: stenosis 50–69%
- Grade 4: stenosis 70–99%
- Grade 5: occlusion

Due to the comparably small lumen, we used a simplified classification scheme for the IMA with grades 1 and 2 and grades 3 and 4 grouped into one category:

- Grade 0: no stenosis
- Grade 1–2: stenosis < 50%
- Grade 3–4: stenosis 50–99%
- Grade 5: occlusion

Significant VAI was defined as at least one stenosis grade 3 or higher (≥ 50%) in any visceral artery (celiac trunk, AMS, AMI or renal artery). For the sub-analysis of renal artery involvement, renal artery involvement was defined as at least one stenosis grade 3 or higher (≥ 50%) in any renal artery. If accessory renal arteries were present, the renal or accessory renal artery with the highest degree of stenosis was reported.

### Statistical analysis

GraphPad Prism 5 was used for statistical analysis. Patient characteristics as well as the frequency and location of visceral artery involvement were analyzed by using descriptive statistics. Continuous data (age and BMI) were found to not be normally distributed using the Shapiro–Wilk test. Therefore, continuous data were displayed as median and range and comparison was performed wit the nonparametric Mann–Whitney test. Categorical data were presented as absolute frequencies and proportions. Frequency distribution of binary data between visceral or renal artery involvement ≥ grade 3 and < grade 3 was compared by using Fisher’s exact test. Distribution of Fontaine stage IIa, IIb, III and IV of patients with PAD was assessed by the chi-square test for trend. *p* values of < 0.05 were defined as statistically significant.

## Results

### Patient characteristics

All 145 patients in the cohort had either known (n = 133, 92%) or suspected PAD (n = 12; 8%). The majority was male with 77% (n = 111). The median age was 68 years ranging from 27 to 91 years. The prevalence of cardiovascular risk factors was high with hypertension (57%) being the most frequent, followed by hyperlipidemia (42%), diabetes (37%) and smoking (31%). Substantial cardiovascular comorbidity was found in the study cohort including documented history of coronary artery disease in 27% of patients and stroke in 8%. Please find a summary of the patient characteristics in Table 1.

**Table 1:** Characteristics of study population

	All patients (n = 145)		VAI ≥ grade 3* (n = 72)			VAI < grade 3* (n = 73)		*p* value
	n	%	n		%	N	%	
Females	34	23%	15		21%	19	26%	0.5573
Age in years, median (range)	68 (27–91)		69 (42–88)			68 (27–91)		0.2578
BMI in kg/m^2^, median (range)	27 (17–53)		26 (18–37)			28 (17–53)		0.9330
CRP in mg/L, median (range)	8.565 (0–289)		8.860 (0–289)			8.490 (0–234)		0.7181
Indication for MRA								
Suspected PAD	12	8%	6	8%		6	8%	1.0000
Known PAD	133	92%	66	92%		67	92%	
Patients with information on Fontaine stage of PAD (n = 121)								
Fontaine stage IIa	3	2%	2	3%		1	2%	0.2535
Fontaine stage IIb	64	53%	35	58%		29	48%	
Fontaine stage III	10	8%	3	5%		7	11%	
Fontaine stage IV	44	36%	20	33%		24	39%	
Cardio-vascular risk factors								
Smoking	45	31%	26	36%		19	26%	0.2122
Diabetes	54	37%	27	38%		27	37%	1.0000
Arterial hypertension	83	57%	44	61%		40	55%	0.5022
Hyperlipidaemia	61	42%	25	35%		36	49%	0.0930
Vascular comorbidities								
Coronary artery disease	39	27%	22	31%		16	22%	0.2616
Cerebrovascular disease	12	8%	6	8%		5	7%	0.7646

VAI ≥ grade 3 = visceral artery involvement with diameter stenosis ≥ 50%

*BMI* body mass index, *MRA* magnetic resonance angiography, *PAD* peripheral artery disease

### Visceral artery involvement in patients with PAD

**Fig. 1:**
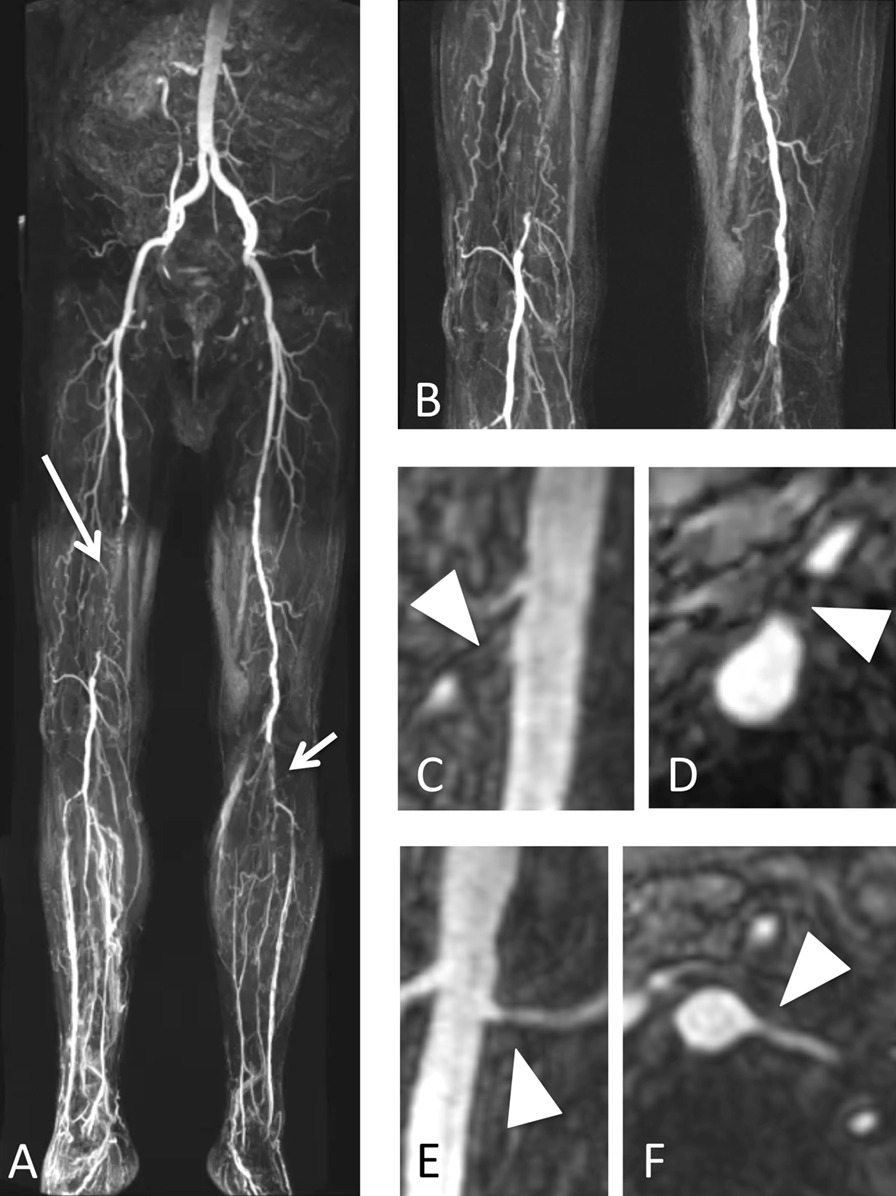
Run-off MR angiography in a 74-year old male patient with known Stage IV peripheral artery disease. Subtracted images post contrast show occlusion of the distal portion of the right superficial femoral artery (long arrow in **a**, magnified in **b**) and of the left popliteal artery segment P3 (short arrow in **a**, magnified in **b**). T1-weighted post-contrast images of the visceral arteries demonstrate an occlusion of the superior mesenteric artery in the sagittal and oblique transverse plane (arrowhead in **c** and **d**) and a mild stenosis of the left renal artery, which was classified as grade 2, in the coronal and oblique transverse plane (arrowhead in **e** and **f**)

The celiac trunk was visualized in 116 (80%) of the patients, the SMA in 131 (90%), the left renal artery in 144 (99%), the right renal artery in 142 (98%) and the IMA in all 145 patients on MRA. Absence of left or right renal artery was detected on MRA in four patients (3%) who had undergone radical nephrectomy. VAI with a stenosis of at least 50% (VAI ≥ grade 3) was detected in 50% of patients (n = 72, 79% men (n = 57) and 21% women (n = 15), Fig. 1). For a detailed description of the prevalence of visceral artery involvement, please see Table 2. There were no differences in age, gender, BMI, CRP levels, indication for MRA, Fontaine stage of PAD, cardiovascular risk factors or vascular comorbidities between patients with and without significant visceral artery involvement on MRA (Table 1). Additional analyses were performed comparing tertiles of age groups ranging from 27 to 60 years (median of 56 years), 61–73 years (median of 68 years) and 74–92 years (median of 77 years) regarding visceral artery involvement, but revealed no significant difference (see supplementary Table 1).

**Table 2:** Detailed prevalence of visceral artery stenosis

Artery	Grade 0		Grade 1		Grade 2		Grade 3		Grade 4		Grade 5	
	n	%	n	%	n	%	n	%	n	%	n	%
CT (n = 116)	34	29	20	17	23	20	22	19	14	12	3	3
SMA (n = 131)	71	54	20	15	19	15	11	8	8	6	2	2
Left RA/ARA* (n = 144)	98	68	19	13	7	5	5	3	10	7	5	3
Right RA/ARA* (n = 142)	89	63	19	13	17	12	6	4	7	5	4	3

	Grade 0		Grade 1–2		Grade 3–4		Grade 5	
	n	%	n	%	n	%	n	%
IMA (n = 145)	97	67	28	19	9	6	11	8

The stenosis with the highest grade is reported in patients who also have accessory renal artery/arteries in addition to the renal artery on the same side

*CT* celiac trunk, *SMA* superior mesenteric artery, *RA* renal artery, *ARA* accessory renal artery, *IMA* inferior mesenteric artery

CT, SMA, RA and ARA: grade 0: no stenosis; grade 1: stenosis < 30%; grade 2: stenosis 30–49%; grade 3: stenosis 50–69%; grade 4: stenosis 70–99%; grade 5: occlusion

IMA: grade 0: no stenosis; grade 1–2: stenosis < 50%; grade 3–4: stenosis 50–99%; grade 5: occlusion

### Sub-analysis of renal artery involvement

**Fig. 2:**
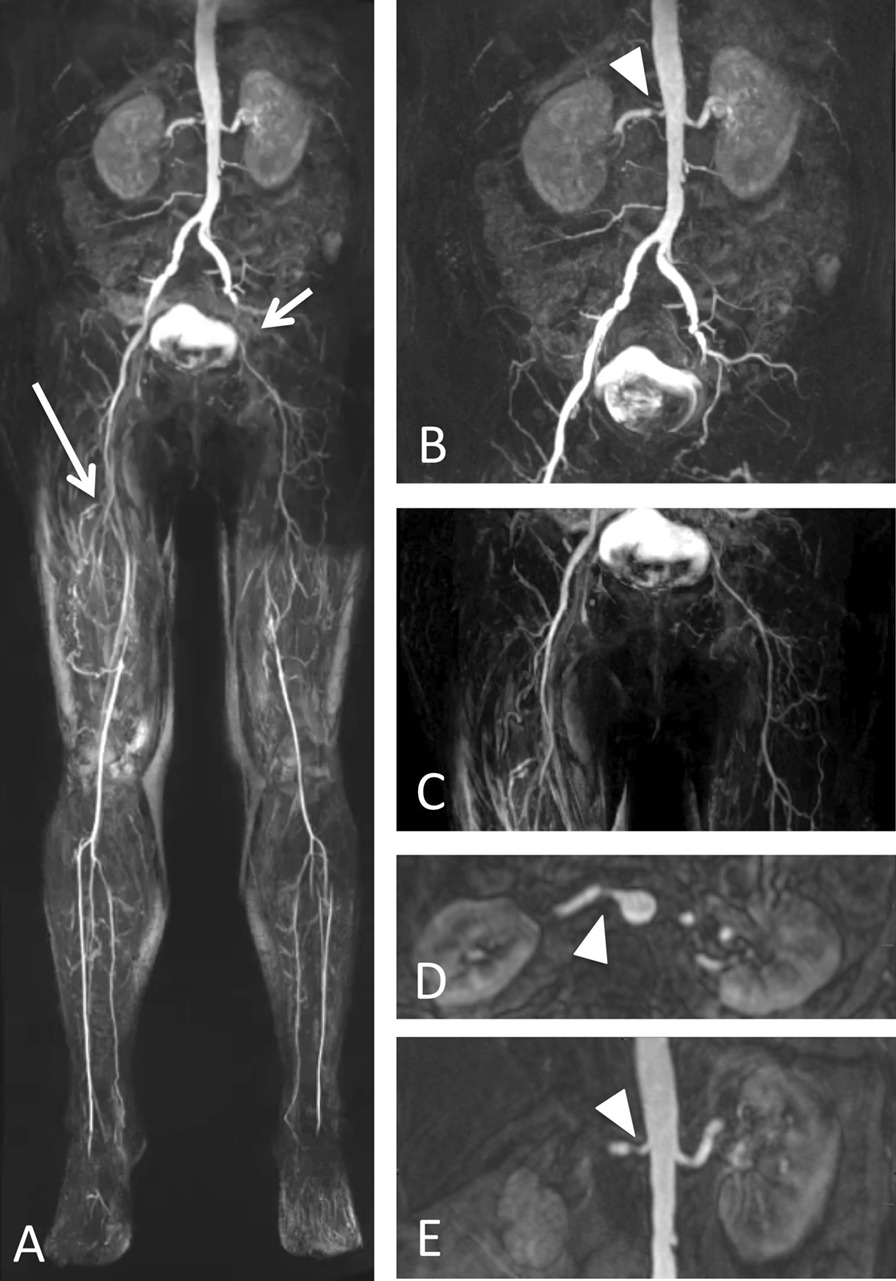
Run-off MR angiography in a 69-year old woman with known Stage IIb peripheral artery disease and hypertension. Subtracted images post contrast show occlusion of the proximal right superficial femoral artery (long arrow in **a**, magnified in **c**), the left external iliac artery and proximal left superficial femoral artery (short arrow in **a**, magnified in **b**). Subtracted image of the abdomen and 3D T1-weighted images post contrast of the visceral arteries demonstrate a stenosis of the right renal artery (arrowhead in **b**, **d** and **e**), which was classified as grade 3

Sub-analysis of 141 patients was performed to evaluate the clinical significance of renal artery involvement. Significant renal artery involvement (≥ 50% diameter stenosis) was observed in 20% of patients (n = 28, Fig. 2). Patients with significant renal artery involvement were more likely to suffer from hypertension (79 vs. 54%, *p* = 0.019) and impaired renal function (estimated glomerular filtration rate by Cockroft–Gault 70 vs. 88 mL/min/1.73 m^2^, *p* = 0.0137, Table 3). There were no differences in gender, age, serum creatinine, CRP levels, diabetes, indication for MRA, Fontaine stage of PAD or presence of accessory renal artery between patients with and without renal artery involvement ≥ grade 3 on MRA.

**Table 3:** Involvement of renal arteries

	All patients (n = 141)			RAI ≥ grade 3 (n = 28)		RAI < grade 3 (n = 113)		*p* value
	n	%		n	%	n	%	
Females	33	23%		9	32%	24	21%	0.2233
Age in years, median (range)	68 (27–91)			67 (52–85)		68 (27–91)		0.7781
eGFR in mL/min/1.73 m^2^, median (range), (n = 134)	84 (25–253)			70 (25–138)		88 (30–253)		**0.0137**
Creatinine in μmol/L median (range), (n = 134)	83 (12–205)			95 (50–205)		82 (12–158)		0.0903
CRP in mg/L, median (range)	8.490 (0–289)			9.375 (0–55)		8.270 (0–289)		0.8483
Arterial hypertension	83		59%	22	79%	61	54%	**0.0192**
Diabetes	54		38%	10	36%	44	39%	0.8303
Indication for MRA								
Suspected PAD	12		9%	1	4%	11	10%	0.4599
Known PAD	129		91%	27	96%	102	90%	
Fontaine stage IIa	3		3%	0	0%	3	3%	0.0684
Fontaine stage IIb	61		52%	18	72%	43	47%	
Fontaine stage III	10		9%	2	8%	8	9%	
Fontaine stage IV	43		37%	5	20%	38	41%	
Presence of accessory renal artery	24		17%	6	21%	18	16%	0.5742

*p* values < 0.05 appear bold

*RAI* renal artery involvement, *eGFR* estimated glomerular filtration rate by Cockroft–Gault, *MRA* magnetic resonance angiography, *PAD* peripheral artery disease

## Discussion

The prevalence of visceral artery involvement in a cohort of patients with known or suspected PAD and leg symptoms examined with run-off MRA has not been described previously. Significant visceral artery involvement with diameter stenosis ≥ 50% on run-off MRA was found to be very common in this population, affecting half of our study cohort (72 of 145 patients). Renal artery involvement with ≥ 50% diameter stenosis was observed in 20% of the patients (28 of 141 patients). Patients with significant renal artery involvement were more likely to suffer from hypertension and impaired renal function.

The prevalence of renal artery involvement seen on MRA in our cohort is comparable with the results of earlier studies using digital subtraction angiography, ranging from 14 to 26% [11, 12, 21]. These prior studies reported a significant association between renal artery stenosis and lower renal function or higher rates of hypertension [11, 12, 21] similar to our study. Only one previous study observed not only on the prevalence of renal but also visceral artery involvement in patients with PAD [22]. This prior study found calcification of the coeliac axis and/or SMA and/or renal arteries in 62% of patients (n = 89) with PAD Fontaine stage 3 or 4. However in this prior study, visceral artery calcification was quantified on CT images and degree of visceral artery stenosis was not determined.

Our data demonstrated no significant difference in the prevalence of diabetes, hyperlipidemia, smoking, obesity or cardiovascular comorbidities in PAD patients with and without visceral artery involvement. Similar results have been reported in patients with coronary artery disease undergoing cardiac catherization [23, 24]. However in these studies, patients with visceral artery involvement were significantly older than patients without visceral artery involvement, whereas we did not identify any age related difference in our study. This inconsistency may be due to the different patient cohorts (peripheral versus coronary artery disease) with both peripheral artery disease of the legs and visceral arteries possibly occurring later in atherosclerotic disease than coronary artery disease [18, 25, 26].

Importantly, we describe the prevalence of incidental visceral artery involvement in patients with known or suspected PAD, who were examined with MRA due to leg symptoms, not due to suspected visceral artery disease. However, stenosis of the visceral arteries can be associated with a broad constellation of clinical disorders and ultimately with an increased mortality risk [12, 18]. Clinical symptoms of VAI can be nonspecific, including post-prandial abdominal pain and weight loss, but ultimately lead to severe complications such as for instance acute mesenteric ischemia and bowel necrosis [23]. Moreover, renal artery involvement cannot only result in kidney failure, but also arterial hypertension and consequently more vascular damage. This vicious circle underscores the importance of an early detection of VAI [19]. Atherosclerosis is a systemic disease and VAI represents progression of the same disease process that leads to PAD of the lower extremities [18]. Although MRA, a commonly performed diagnostic test in patients with PAD, frequently visualizes the visceral arteries, MRA scoring systems for patients with PAD, such as the run-off resistance score [16] and MRA index [17], do not take into account visceral artery involvement.

The results of our investigation should be interpreted in light of its limitations. The MRI protocol may not have been optimal for the detection of VAI, since the examination was focused on the evaluation of the peripheral vascular system. In particular, the celiac trunk and the superior mesenteric artery were visualized in most but not all patients. Our results might therefore underestimate the true extent of visceral artery involvement in patients with known or suspected PAD. An external reference standard, e.g. digital subtraction angiography (DSA), was not available for validation of the diagnosis and degree of visceral artery stenosis. However, 3D gadolinium-enhanced MRA has previously been shown to have high sensitivity and specificity in evaluating the proximal coeliac, superior mesenteric [27, 28] and renal arteries. For example the sensitivity for detection of renal artery stenosis with DSA as the gold standard method has shown to be 90% for MRA, 94% for Computed Tomography Angiography and 75% for Doppler ultrasound [27, 29]. Finally, estimated glomerular filtration rate was calculated by using the Cockroft–Gault formula, which does not account for body surface area and may underestimate high GFR in older age groups [30].

In conclusion, run-off MRA in patients with PAD and leg symptoms often visualizes the visceral arteries in addition to the peripheral vasculature. Visceral artery involvement is very commonly seen in this patient population. Finding of visceral artery involvement on run-off MRA obtained for PAD evaluation can be used to inform risk stratification. This may be particularly relevant in patients with hypertension and impaired renal function, since they are more frequently affected by renal artery involvement in addition to PAD of the lower extremities.

## Supplementary Information

**Additional file 1: Supplementary Table 1**. Visceral artery stenosis comparing tertiles of age groups.

## Data Availability

The datasets used and analyzed during this study are available from the corresponding author on reasonable request.

## Abbreviations

GFR: glomerular filtration rate
IMA: inferior mesenterial artery
MRA: magnetic resonance angiography
PAD: peripheral artery disease
SMA: superior mesenterial artery
TWIST: time-resolved angiography with interleaved stochastic trajectories
VAI: visceral artery involvement
RA: renal artery

## Declarations

### Previous presentations at scientific conferences

This article was previously presented as an electronic poster at the 2020 IROS (Interventionell Radiologisches Olbert Symposium) on January 16–18, 2020 in Salzburg [31].
