# Supplementary material for: Prevalence of visceral artery involvement in patients with peripheral artery disease found on run-off MRA
Source: BMC Med Imaging. 2021 Jun 2;21:93. doi: 10.1186/s12880-021-00615-2 (PMC8171056; doi:10.1186/s12880-021-00615-2)
Supplement: Supplementary file 1 — Additional file 1: Supplementary Table 1. Visceral artery stenosis comparing tertiles of age groups. [file 12880_2021_615_MOESM1_ESM.docx]

**Supplementary Table 1: Visceral artery stenosis comparing tertiles of age groups**

| Age groups | 27 to 60 years | | 61 to 73 years | | 74 to 92 years | | p-value |
| --- | --- | --- | --- | --- | --- | --- | --- |
|  | **n** | **%** | **n** | **%** | **n** | **%** |  |
| Median age (years) | 56 | | 68 | | 77 | |  |
| VAI ≥ grade 3 | 24 | 49 | 22 | 44 | 26 | 57 | 0.4741 |
| CT ≥ grade 3 | 24 | 49 | 25 | 50 | 18 | 39 | 0.4132 |
| SMA ≥ grade 3 | 14 | 29 | 9 | 18 | 12 | 26 | 0.4005 |
| RAI ≥ grade 3 | 11 | 22 | 6 | 12 | 11 | 24 | 0.4158 |
| IMA ≥ grade 3 | 8 | 16 | 5 | 10 | 7 | 15 | 0.5926 |

Grade 3 = Artery with diameter stenosis ≥ 50%

VAI: visceral artery involvement; CT: celiac trunk; SMA: superior mesenteric artery; RA: renal artery; ARA: accessory renal artery; IMA: inferior mesenteric artery.
